# The impact of meaning in life and professional happiness on the turnover intention of health care workers: a cross-sectional study from China

**DOI:** 10.1186/s12960-023-00878-6

**Published:** 2023-11-27

**Authors:** Yuting Huang, Huilin Zhang, Zuming Qin, Ying Zou, Zhiling Feng, Jiao Cheng

**Affiliations:** 1 Clinical Nursing Teaching and Research Section, Second Xiangya Hospital, Central South University, Changsha, Hunan China; 2 Present Address: Xiangya School of Nursing, Central South University, Changsha, Hunan China; 3 Present Address: School of Nursing, Hunan University of Chinese Medicine, Changsha, Hunan China; 4 Trade Union, Second Xiangya Hospital, Central South University, Changsha, Hunan China; 5 Youth League Committee, Second Xiangya Hospital, Central South University, Changsha, Hunan China

**Keywords:** Health care workers, Turnover intention, Professional happiness, Meaning in life, Positive psychology, Health human resources

## Abstract

**Introduction:**

The turnover and shortage of health care workers (HCWs) have been a worldwide problem for healthcare organizations. The primary aim of this study was to identify the factors influencing the intention of Chinese HCWs to leave their job, especially meaning in life and professional happiness.

**Methods:**

This observational cross-sectional study, conducted among 1125 full-time HCWs, assessed demographic variables, meaning in life, professional happiness, and turnover intention by a survey. The survey was distributed to HCWs in three tertiary hospitals. The data were analyzed by T-tests, ANOVA, Kruskal–Wallis tests and hierarchical linear regression model.

**Results:**

There were statistically significant differences in turnover intention of HCWs by gender, age, role, educational level, years in practice, and number of monthly night shifts. HCWs’ meaning in life and professional happiness were negatively associated with the turnover intention. Furthermore, after controlling for other factors, meaning in life explained 3.7% of the turnover intention and professional happiness explained 13.4%.

**Conclusion:**

In our study, positive psychological factors were related to turnover intentions. Professional happiness was the strongest predictor. Thus, health human resource managers should foster positive psychology among HCWs to reduce their turnover.

**Supplementary Information:**

The online version contains supplementary material available at 10.1186/s12960-023-00878-6.

## Background

Turnover seems to be inevitable in human resource management, signifying the flow of personnel within an organization [1]. High turnover can lead to increased costs and difficulty in management, and decreased morale [2]. It becomes a prevalent concern within the healthcare sector, where healthcare workers (HCWs) face increased workloads, reduced wages, and challenging working conditions [3]. Notably, the results of a global systematic review showed a 47.0% turnover rate for general practitioners (GPs) [4]. The turnover rate for hospital nursing staff was 41.3% [5]. In China, 70.0% of GPs highly intended to leave [6]. Even under the health personnel incentive mechanism reform, the turnover intention rate of HCWs in Shanghai still reached 15.8% [7]. Consequently, the turnover of HCWs can profoundly impact hospital profits. For example, a recent report in the US highlighted that every percentage point increase in nurse turnover costs hospitals an average of $52,350 [8]. Furthermore, the turnover and shortages of HCWs have received significant attention as a global issue, affecting the performance of healthcare organizations.

Turnover intention is defined as the probability that the organization’s employees will leave voluntarily at some point in the near future [9]. It is about an individual’s vision of a possible departure and is considered to be the strongest predictor of actual departure of medical staff [10]. The individual’s intention to leave may reflect organizational management problems, such as low work status, absenteeism and poor performance, and understanding it may help organizations find ways to prevent or reduce actual staff departures. Therefore, the turnover intention is the focus of this study. Importantly, investigations have shown that employee turnover is associated with low incomes [11], poor working conditions [12], high job-related stress [13], and frequent workplace violence [14]. However, little research explores the psychological role of employee turnover.

Positive psychology advocates a positive orientation in psychology, studying the positive psychological qualities of human beings and focusing on their health, well-being, and harmonious development [15]. Additionally, the HCWs’ positive psychological state helps nurture and effectively manage their psychological potential, thus enhancing their work performance. However, most scholars focused on employee inefficiency and organizational dysfunction rather than employee potential and positive strengths, especially among HCWs [16–19]. Moreover, meaning in life and professional happiness are two important components of positive psychology. The former means that an individual finds the goal of life by reflecting on their reason or purpose of existence. It has a moderating effect on mental health in stressful conditions and is essential for personal survival in adversity [20]. In addition, it is associated with positive emotions, life satisfaction and career decision-making self-efficacy [21]. Professional happiness, a positive energy effect of employees in their work, is a feeling about the occupation generated by realizing their own value and emotion. It is the psychological satisfaction of employees directly obtained from work-related perspectives, such as work content, environment, and remuneration. Although this concept is relatively new, there is evidence that investing in professional happiness in organizations can lead to success [22]. In summary, this study argues that more positive psychology (e.g., meaning in life and professional happiness) negatively predicts the turnover intention of HCWs.

Therefore, this study aimed to explore HCWs’ meaning in life and professional happiness and the impact of the two psychological components on HCWs’ turnover intention. For this purpose, a cross-sectional survey was conducted in three hospitals in Hunan, China. Furthermore, the factors influencing turnover need to be better understood by district health managers, who are critical at the health system’s operational level and essential performance drivers in their districts.

## Methods

### Study design, sampling and participants

This study was designed as an observational cross-sectional study. It included HCWs in three level A tertiary hospitals in Hunan Province from March 2023 to April 2023. Hospitals are classified into 3 categories in China: primary, secondary, and tertiary (the highest). Moreover, based on their capability to provide medical care, medical education, and to conduct medical research, according to the total score of 1000 points, hospitals are divided into level A (the highest), B, C and so on. Tertiary hospitals (with more than 501 beds) provide medical and health services across regions, provinces, cities, and the country, providing high-level specialized medical and health services and carrying out higher education and scientific research. Level A is graded for more than 900 scores. Notably, level A tertiary hospitals are the highest-level hospitals in China. The personnel requirements list is as follows: (1) at least 1.03 health technicians per bed; (2) at least 0.4 nurses per bed; (3) the director of each department with the title of deputy chief physician or above; (4) no less than 2 clinical nutritionists; (5) no less than 1% of engineering technicians. For example, in a level A tertiary hospital in Hunan Province, the number of outpatient and emergency services was 4.152 million, with 175,000 discharged patients and 142,000 surgeries in 2022. The hospital now has 5301 employees, including 2558 registered nurses.

Firstly, cluster random sampling was used. The random numbers were generated for each of the 57 tertiary hospitals in the Hunan Province by computer, and the three hospitals with the smallest random numbers were selected for the survey. Secondly, convenience sampling was applied to recruit volunteers for the questionnaire from the three hospitals’ clinical departments, medical and technical departments, research centers and administrative departments.

This study was conducted according to strict inclusion and exclusion criteria for the survey participants. The inclusion criteria list as follows: (1) HCWs, (2) full-time job in level A tertiary hospitals, (3) in normal physical and mental condition, and (4) volunteers to participate in this survey. The exclusion criteria included interns who had not yet obtained their practicing certificates. Before completing the questionnaire, each participant received an online training for 15–25 min, including the survey’s purpose, content, process, and precautions.

The study collected data through an online questionnaire platform (https://www.wjx.cn/). The project manager was responsible for contacting hospital nursing directors, who assisted hospitals in recruiting volunteers, completing training and distributing the questionnaire. In the process of filling in the questionnaire, if there were any missing information, a “prompt” to fill in the missing part would appear after the participants clicked on “submit”; they would not be able to successfully submit until they had finished all the information, thus ensuring the completeness of the questionnaire. The backend was set to allow each respondent to answer the questionnaire only once to avoid repeating the questionnaire. Two researchers from the team monitored the data in real-time and exported the data when no new data were generated for a week. Another two researchers double-checked the quality of the questionnaires and deleted any short questionnaires that violated the requirements or had contradictory answers. During the survey period, 1214 questionnaires were collected, of which 1125 were valid, giving a reasonable questionnaire return rate of 92.67%. Furthermore, the authors followed the Strengthening the Reporting of Observational Studies in Epidemiology (STROBE) reporting checklist for cross-sectional studies.

### Sample estimation

For the regression analysis, the sample size was calculated using N ≥ 20*m (m = the number of independent variables) to verify validity. Our study tested 46 independent variables in each hospital, so the minimum sample size was 920. Considering a 15% attrition rate, we estimated a larger sample size, i.e., a minimum of 1083 HCWs were required for this study. Thus, the sample size for this study was adequate.

### Ethical consideration

Participants were informed of the purpose of the study, that their participation was voluntary and that they had the right to terminate their participation at any time. Before answering the questionnaire, participants had to read and agree to the informed consent form. The questionnaires were anonymous, and the study data were stored in an encrypted computer that only the researcher could access. This study has been approved by the Ethical Review Committee of Nursing and Behavioral Medicine Research, School of Nursing, Central South University (Approval No. E202361).

### Outcome measures

#### Personal and professional characteristics

This included gender, age, marital status, role, educational level, years in practice, professional title, income (monthly), and number of night shifts (monthly).

#### Meaning in life

The Chinese version of the meaning in life questionnaire (MLQ) was used in this study. This scale was developed by Steger in 2006 [23], and translated into Chinese by Mengcheng W in 2008 [24]. It is used to assess the level of meaning in life. The scale contains 10 items, divided into two dimensions—the search for meaning (MLQ-S) and the presence of meaning (MLQ-P), with 5 items in each dimension. Each item is rated on a 7-point Likert scale, with a total score ranging from 10 to 70. Higher scores indicate a higher level of life meaning. Moreover, the Cronbach’α coefficients for each dimension and the total scale are 0.82 to 0.85 [24].

#### Professional Happiness

The Professional Happiness Scale for Medical Workers was developed by Dongmei H in 2011 [25], which measures medical workers’ happiness at work. The scale consists of 24 items in 5 dimensions, including 6 items on physical and mental health, 6 items on value or ability, 5 on social support, 3 on income, and 4 on work environment. Each item is assessed on a 5-point Likert scale ranging from 1 (not at all) to 5 (fully), and the total score ranges from 24 to 120. The higher the score, the greater the happiness. The Cronbach’α coefficients for each dimension and the total scale are 0.84 to 0.97 [25].

#### Turnover Intention

The Turnover Intention Scale used in this study was developed by Michael and Spector in 1982 [26]. It was translated and revised into Chinese version by Don-Yon L and Gin-Yuan L [27]. Xinbin F [28] adapted the scale for use among HCWs. The scale consists of three questions. Each item is assessed using a 4-point Likert scale ranging from 1 (never) to 4 (often), and the total score ranges from 3 to 12. Higher scores indicate a higher likelihood of quitting one’s current job. Based on China’s national condition, the scale has been widely accepted as a measure of assessing Chinese HCWs’ willingness to leave their jobs. It has also shown high reliability among them. The Cronbach’α coefficient of the scale is 0.878 [28].

### Data analysis

Data analysis was conducted with SPSS 26.0. Descriptive statistics were used to describe the demographic characteristics, meaning in life, professional happiness, and turnover intention among HCWs. Differences in turnover intention between demographic characteristics were examined by t-tests, one-way analysis of variance (ANOVAs), or Kruskal–Wallis tests. LSD and Bonferroni correction were used for multiple comparisons. Furthermore, the Pearson correlation analysis was applied to test the correlation between meaning in life, professional happiness, and turnover intention.

The independent variables with *P* < 0.1 in the one-way analysis of variance and correlation analysis were included in a hierarchical linear regression analysis, with the turnover intention as the dependent variable. The independent variables were entered in three steps: the first step was adding control variables, such as gender, age group, and marital status, to the model; meaning in life was added in the second step; and happiness was added in the third step. All differences were tested using a two-tailed test, and the significance level was set at *P* < 0.05.

## Results

### Characteristics and univariate analysis of HCWs

Of the 1125 respondents in the three tertiary hospitals, 84.7% were female. The mean age of the participants was (30.64 ± 4.647) years. Most were nurses, married, and had obtained a bachelor’s degree. From univariate analysis, there were statistically significant (*p* < 0.05) differences in turnover intention in terms of gender, age, role, educational level, years in practice, and number of night shifts (monthly) (Table 1).

**Table 1:** Characteristics and univariate analysis of the turnover intention (n = 1125)

Characteristics	Participants, no. (%)	Turnover intention (M ± SD)	*t/F/Z*	*P*
Gender				
Male	172 (15.3)	6.96 ± 2.078	− 2.331^t^	0.020
Female	953 (84.7)	7.32 ± 1.847		
Age group				
< 30	456 (40.5)	7.45 ± 1.855	3.974^F^	0.019
30–40	657 (58.4)	7.15 ± 1.893		
41–51	11 (1.1)	6.67 ± 2.387		
Marital status				
Unmarried	424 (37.7)	7.42 ± 1.874	2.456^F^	0.086
Married	686 (61.0)	7.19 ± 1.889		
Divorced	15 (1.3)	6.80 ± 2.042		
Role				
Physician	129 (11.5)	7.70 ± 1.818	41.809^Z^	< 0.001
Nurse	779 (69.2)	7.40 ± 1.763		
Laboratory or radiology technician	112 (10.0)	6.61 ± 2.072		
Researcher (without clinical role)	10 (0.9)	7.30 ± 2.058		
Administrative	95 (8.4)	6.36 ± 2.269		
Educational level				
Associate degree or lower	26 (2.3)	7.35 ± 1.765	3.734^F^	0.011
Bachelor’s degree	767 (68.2)	7.34 ± 1.819		
Master’s degree	216 (19.2)	6.89 ± 1.976		
Doctor’s degree or higher	116 (10.3)	7.46 ± 2.116		
Years in practice				
≤ 5	394 (35.0)	7.31 ± 1.919		
6–10	472 (42.0)	7.52 ± 1.757		
11–15	171 (15.2)	6.95 ± 1.977		
16–20	69 (6.1)	6.48 ± 1.720		
> 20	19 (1.7)	5.79 ± 2.440		
Professional title				
Primary title	503 (44.7)	7.38 ± 1.895		
Middle title	598 (53.2)	7.18 ± 1.863		
Vice-senior title	20 (1.8)	7.10 ± 2.532		
Senior title	4 (0.4)	7.25 ± 0.500		
Income (CNY)				
≤ 6000	219 (19.5)	7.47 ± 2.005	2.042^F^	0.130
6001–9000	408 (36.3)	7.29 ± 1.830		
> 9000	498 (44.3)	7.16 ± 1.878		
Number of night shifts (monthly)				
None	205 (18.2)	6.55 ± 2.152	47.300^Z^	< 0.001
< 5	315 (28.0)	7.19 ± 1.892		
5–9	358 (31.8)	7.46 ± 1.791		
10–15	194 (17.2)	7.58 ± 1.628		
> 15	53 (4.7)	8.08 ± 1.426		

An associate degree requires 3 years of education in college after graduation from senior middle school (grades 10 to 12) or 5 years of education in college after graduation from junior middle school (grades 7 to 9). CNY: Chinese Yuan

^t^T-test

^F^One-way analysis of variance

^Z^Kruskal–Wallis test

### Descriptive analysis

Table 2 shows the descriptive data for the study variables. The turnover intention score was 7.27 (SD = 1.888). The meaning in life score was 49.96 (SD = 8.499), with a score of 24.15 (SD = 4.464) for the presence of meaning and 25.81 (SD = 5.194) for the search for meaning. Moreover, the total professional happiness score was 78.86 (SD = 15.256), with the lowest average scores in the physical and psychological health and income dimensions. More than half of the medical staff sometimes (43.5%) or often (10.6%) considered resigning from their jobs, and most would not choose to work in the medical profession again after quitting (Table 3).

**Table 2:** Total scores and scores of various dimensions (n = 1125)

Item	Score range	Mean ± SD
Turnover intention	3–12	7.27 ± 1.888
Meaning in life	10–70	49.96 ± 8.499
The presence of meaning, MLQ-P	5–35	24.15 ± 4.464
The search for meaning, MLQ-S	5–35	25.81 ± 5.194
Professional happiness	24–120	78.86 ± 15.256
Physical and psychological health	6–30	16.67 ± 5.730
Value or ability	6–30	20.42 ± 4.561
Social support	5–25	19.74 ± 3.374
Income	3–15	8.68 ± 2.872
Working environment	4–20	13.36 ± 3.137

**Table 3:** Distribution of each item of turnover intention, participants, no. (%)

Item	Never	Rarely	Sometimes	Frequently
Would you consider resigning?	220 (19.6)	297 (26.4)	489 (43.5)	119 (10.6)
Would you look for the same job after quitting?	337 (33.5)	353 (31.4)	231 (20.5)	164 (14.6)
Would you change jobs of a different nature after quitting?	193 (17.2)	257 (22.8)	420 (37.3)	255 (22.7)

### Bivariate analysis

Table 4 shows the correlation between meaning in life, professional happiness, and turnover intention. Significant correlations were found between all the variables studied. Meaning in life and all its dimensions were positively correlated with professional happiness and negatively correlated with turnover intention. Professional happiness was negatively correlated with turnover intention.

**Table 4:** Variable relevance

	1	2	3	4	5	6	7	8	9
1. Meaning in life	1								
2. The presence of meaning	0.859^**^	1							
3. The search for meaning	0.898^**^	0.546^**^	1						
4. Professional happiness	0.464^**^	0.467^**^	0.358^**^	1					
5. Physical and psychological health	0.278^**^	0.326^**^	0.175^**^	0.770^**^	1				
6. Value or ability	0.435^**^	0.438^**^	0.336^**^	0.861^**^	0.513^**^	1			
7. Social support	0.418^**^	0.396^**^	0.343^**^	0.718^**^	0.360^**^	0.601^**^	1		
8. Income	0.312^**^	0.296^**^	0.256^**^	0.727^**^	0.428^**^	0.579^**^	0.388^**^	1	
9. Working environment	0.383^**^	0.343^**^	0.332^**^	0.767^**^	0.396^**^	0.620^**^	0.532^**^	0.581^**^	1
10. Turnover intention	− 0.218^**^	− 0.250^**^	− 0.142^**^	− 0.452^**^	− 0.403^**^	− 0.364^**^	− 0.304^**^	− 0.316^**^	− 0.316^**^

^**^p < 0.01 (two-tailed)

### Multivariate analysis

Table 5 shows the results of the hierarchical linear regression. Using the turnover intention of HCWs as the dependent variable, the demographic characteristics included in the first step accounted for 7.0% of the turnover intention variance. In the second step, meaning in life accounted for 3.7% of the variance. In the third step, professional happiness accounted for 13.4%. In addition, the turnover intention was influenced by educational level, role as a physician or administrator, and years in practice. By testing the conditions of using this regression model and the multiple cointegration, the results showed that the cointegration diagnostic tolerance of this regression equation ranged from 0.433 to 0.899 (all > 0.1), and the variance inflation factor (VIF) ranged from 1.112 to 2.309 (all < 5). Notably, this indicated no multicollinearity between the variables. Furthermore, the Durbin–Watson test, D = 1.894, demonstrated that the residuals were independent.

**Table 5:** The hierarchical linear regression analysis for turnover intention

	Turnover intention		
	Step1(β)^a^	Step2(β)^a^	Step3(β)^a^
Gender (ref: male)			
Female	0.397^*^	0.339^*^	0.267
Educational level	− 0.201	− 0.164	− 0.232^*^
Number of night shifts (monthly)	0.213^***^	0.190^**^	0.069
Marital status (ref: unmarried)			
Married	− 0.010	0.030	0.093
Divorced	− 0.301	− 0.157	− 0.299
Role (ref: nurse)			
Physician	0.701^**^	0.691^**^	0.654^**^
Laboratory or radiology technician	− 0.353	− 0.384	− 0.205
Researcher (without clinical role)	0.499	0.392	0.291
Administrative	− 0.587^*^	− 0.566^*^	− 0.579^**^
Years in practice	− 0.245^**^	− 0.224^**^	− 0.249^**^
Age group	− 0.046	− 0.077	− 0.117
Meaning in life		− 0.043^***^	− 0.001
Professional happiness			− 0.053^***^
F	8.669	12.179	28.508
Adjusted R^2^	0.070	0.107	0.241
∆R^2^		0.037	0.134

^a^Unstandardized regression coefficient

^*^p< 0.05, ^**^p < 0.01, ^***^p < 0.001 (two-tailed)

## Discussion

Given China’s large population, the heavy workload of HCWs, and the difficulty changing their working environment in the short term, it is significant for this study to focus on the positive psychology of HCWs to reduce turnover. This study explored the impact of Chinese HCWs’ meaning in life and professional happiness on the turnover intention. Meanwhile, we analyzed and controlled for the role of some personal and professional characteristics on turnover intention. The turnover intention score of HCWs in this study was (7.27 ± 1.888), with an average mean score of (2.42 ± 0.629), higher than that of HCWs in Ontario Hospital, Canada [29]. These two studies were conducted at a particular point when COVID-19 was coming to an end and was about to occur. Following a prolonged COVID-19 epidemic, the increased turnover intention may be due to the stressful workload, difficulty in work–life balance, and fear of death during the COVID-19 period [30]. In addition, the Terror Management Theory (TMT) suggests that the awareness of death triggers death anxiety and reflection on the meaning and purpose of life and promotes the perception of self-transcendence [31]. Based on this, it has been found that death anxiety induced by COVID-19 indirectly affects turnover intention by increasing the need for meaningful work. Moreover, this effect decreases as the importance of the medical task increases [32]. In other words, the increased turnover intention may stem from a constructive, self-transcendent mechanism. HCWs aspire to uncover the deeper meaning of life and find fulfillment in their work, recognizing their intrinsic self-worth through their professional endeavors; this seems to be supported by the lower levels of life meaning and professional happiness found by the survey. It also validated the need for this study to be conducted. The study results also provide strategies for Chinese healthcare organizations to reduce staff turnover, improve work behavior and enhance organizational efficacy.

We found it interesting that in the healthcare workplace, female employees showed a higher intention to leave, and other researchers have come up with the same results [33]. Also some studies have concluded that gender does not affect turnover [34]. However, studies in other workplaces suggested that men would show a higher turnover intention [35]. This contradiction may reveal that gender may have a specific role in this regard, but it is not conclusive. No specific studies have examined gender differences in turnover intention and whether such differences exist in actual leaving behavior. Our study results showed no statistically significant difference in the average monthly earnings achieved by male and female medical staff (Z = 0.035, *p* = 0.851). However, female staff had lower professional happiness than males, reflecting in the physical and psychological health dimension and value dimension (Additional file 1: Appendix S1); this was likely related to the higher turnover intention of female staff. Of course, this study’s high proportion of female employees (84.7%) may have somewhat biased results. In addition, employees’ turnover intention likewise showed variability in age, role, education level, and years of experience. Furthermore, the results of the multiple comparisons (Additional file 1: Appendix S2) suggested that the turnover intention was higher among HCWs under 30 years of age and physicians by profession, and was the lowest among those with a master’s degree. Notably, the turnover intention decreased with increasing years of experience. In the theory of organizational justice, it becomes evident that employees engage in a perpetual comparative analysis of the rewards they receive (salary, bonuses, promotions, etc.) against their individual inputs (education, intelligence, and experience, etc.) in social exchange. Organizational justice stands as a pivotal factor influencing the motivation and dedication of employees, particularly those possessing specialized knowledge. Failure to attain relative justice can detrimentally impact organizational behavior, potentially resulting in adverse outcomes such as increased turnover rates. Therefore, while the present findings underscore the importance of hospital managers’ attention to specific employee demographics, such as those under 30 years old and physicians, it equally emphasizes the paramount need for health human resource management to strive for equitable treatment across diverse demographic profiles and job characteristics.

The Job Demands-Resources Model (JD-R Model) suggests that in terms of job characteristics, continuous job demands deplete employees’ energy and increase their burnout and turnover intention. In contrast, job resources motivate employees to engage in their work, increase their sense of organizational commitment, and decrease their turnover intention [36]. The night shifts, a more specific job requirement for HCWs, showed variability in this study. The increase in the turnover intention was statistically significant when the number of night shifts exceeded 15 per month. Other researchers have yielded similar results, suggesting that more than 14 night shifts per month would increase the turnover intention [37]. Unfortunately, we have not yet seen a study that specifies this number. There is no doubt that shift work has negative health outcomes for employees, such as cardiovascular disease [38], depression [39], gastrointestinal dysfunction [40], and shift work disorder, which showed an overall prevalence of 26.5% [41], and even up to 84.0% among nurses [42]. Therefore, health workforce management should focus on shift workers’ physical and mental health. Moreover, it is necessary to have regular testing and minimize the number of night shifts per capita per month. Furthermore, after introducing the meaning in life and professional happiness into the model, respectively, the impact of the number of monthly night shifts on HCWs’ turnover intention decreased and even reached statistical insignificance, which implied that fostering a positive psychology can make medical workers more tolerant of the attrition caused by the job demands. When people find meaning in their lives and feel happy, an otherwise dissatisfying work environment may become less important, thus reducing the negative impact of this experience on turnover [43].

HCWs’ meaning in life is negatively related to the turnover intention. After controlling for other factors, meaning in life explains 3.7% of the turnover intention. In other words, meaning in life may protect HCWs’ intention to leave and is a positive resource for employees’ work behavior. It has been suggested that a greater experience of meaning in life is associated with better physical and mental health [44], which may be a reason why meaning of life can reduce turnover intention. In addition, if HCWs find their work meaningful, they are more intrinsically motivated to fulfill their duties with greater determination, even to perform activities beyond their contractual obligations [45]. However, some studies have found that, at the level of meaning, the low contribution of work to one’s life increases turnover intention, but whether work creates value for others shows no effect [46]. Of course, this does not mean that the value of one’s work is unimportant, but that there are other more relevant variables about which further inquiry is needed. In particular, more evidence needs to be gathered in the group of medical professionals whose professional values are characterized by high levels of dedication.

Professional happiness is another even more important job resource, explaining 13.4% of turnover intention. Happiness is a positive internal experience. Having happy and efficient employees is one of the ideals of healthcare organizations, given its impact on the quality of healthcare services [47]. HCWs with high levels of happiness can better maintain positive emotions at work. They exhibit higher levels of organizational citizenship behavior and lower levels of job burnout. As a result, they are less likely to choose to “pull back”, such as being absent from work or leaving [48]. Moreover, Happiness among employees is associated with their productivity, work safety, and job satisfaction [49]. Meaning is among others also postulated as a central pillar to well-being in Seligman’s PERMA model [50]. Our results do find that HCWs’ professional happiness is related to meaning in life, and that the meaning in life was no longer significant in the hierarchical linear regression model after professional happiness is introduced. In this way, some measures to enhance meaning in life might be beneficial for HCWs’ professional happiness. Moreover, the construct of meaning in life is considered to be relatively stable [51], building lasting happiness. In addition, professional happiness is associated with physical and mental health, value or ability, social support, income, and work environment. Interventions based on positive psychology such as the Three Good Things intervention [52] and workplace-based interventions such as workload and time management [53] have been confirmed to enhance happiness. However, given the many factors involved in professional happiness, it is important to think systemically, specifically, to promote HCWs happiness through a holistic and cross-sectoral approach. By developing and institutionalizing policies and programs aimed at improving the happiness of HCWs, it is more likely that optimal performance will be achieved and health workforce will be strengthened.

In a word, meaning in life and professional happiness is not only a supportive resource for work behavior (a positive psychology perspective), but also a protective resource against damage to work behavior (a stress psychology perspective). Stress at work is difficult to avoid altogether, but when HCWs experience meaning in their lives and professional happiness, they also place a high value on their work. Considering positive mental states as a potentially protective resource, creating meaning and happiness allows people the flexibility to respond to and adapt to stress. Therefore, it is crucial that managers need to ensure that their staff do not lack meaning and happiness. To stabilize the workforce, managers should focus on the positive psychological state of HCWs and develop measures to enhance the sense of meaning and positive well-being in work, such as building positive attitudes towards death through death education to help employees determine their meanings and goals in life [54]. Using percussion instruments or music played by individuals or groups can also help relieve work stress and enhance the subjective feeling of professional happiness [55].

## Strengths and limitations

This study is the first to assess the impact of the sense of meaningful life and professional happiness on Chinese HCWs’ turnover intention. Medical workers’ turnover intention and its predictors were investigated from a positive psychology perspective. The analysis found that after controlling for several personal and professional characteristics that are difficult to change, positive psychological states remained a significant predictor of HCWs’ turnover intention. Therefore, this study is valuable for policymakers worldwide who wish to address employment instability in the healthcare sector in their countries. It is particularly relevant in developing countries with constraints in improving the healthcare work environment.

However, this study must consider some limitations when interpreting and applying the findings. Firstly, although the sample size of this study was large, the study population was sourced from Hunan, China and the study sample is predominantly composed of nurses. Therefore, the sample’s representativeness was somewhat limited, making it challenging to represent the overall situation of all medical workers in China. Thus, researchers in the future could conduct multi-center studies and select appropriate sampling methods for the survey. Secondly, although the definition of turnover intention is objective and accurate, and some studies confirmed the correlation between turnover intention and actual leaving behavior [56], the turnover intention obtained through questionnaires may be low [4].

## Conclusion

How to prevent and reduce medical staff turnover has become an important element of human resource management in health care organizations. The personal and occupational characteristic factors influencing HCWs’ turnover intention should be identified. Additionally, the analysis of these factors and positive work-related psychological states highlights the link between meaning in life and professional happiness with HCWs’ turnover intention. Accordingly, positive psychological factors in healthcare workers are beneficial in reducing turnover, with professional happiness being a more significant predictor of turnover than meaning in life. Therefore, effective measures of cultivating HCWs’ positive mindset can be taken to reduce turnover.

### Supplementary Information

**Additional file 1:** **Appendix S1: Table S1.** Meaning in life and professional happiness by gender. **Appendix S2: Table S2.** Multiple comparison by age groups. **Table S3.** Pairwise comparison by role. **Table S4.** Multiple comparison by educational level. **Table S5.** Pairwise comparison by years in practice. **Table S6. **Pairwise comparison by number of night shifts.

## Data Availability

The dataset presented in this article is not readily available due to privacy concerns. Requests to access the datasets should be directed to the corresponding author at 275,143,435@qq.com or 109,055,862@qq.com.

## Abbreviations

HCWs: Health care workers
GPs: General practitioners
MLQ: The meaning in life questionnaire
MLQ-S: The search for meaning
MLQ-P: The presence of meaning
CNY: Chinese Yuan
VIF: Variance Inflation Factor
Ref: Reference
JD-R Model: Job Demands-Resources Model
