# Supplementary material for: The impact of meaning in life and professional happiness on the turnover intention of health care workers: a cross-sectional study from China
Source: Hum Resour Health. 2023 Nov 27;21:92. doi: 10.1186/s12960-023-00878-6 (PMC10680312; doi:10.1186/s12960-023-00878-6)
Supplement: Supplementary file 1 — Additional file 1: Appendix S1: Table S1. Meaning in life and professional happiness by gender. Appendix S2: Table S2. Multiple comparison by age groups. Table S3. Pairwise comparison by role. Table S4. Multiple comparison by educational level. Table S5. Pairwise comparison by years in practice. Table S6. Pairwise comparison by number of night shifts. [file 12960_2023_878_MOESM1_ESM.docx]

Appendix S1

| **Table S1 Meaning in life and professional happiness by gender** | | | | | | |
| --- | --- | --- | --- | --- | --- | --- |
|  | Gender | n | mean | Standard deviation | t | *P* |
| Meaning in life | Male | 172 | 51.07 | 8.837 | 1.861 | 0.063 |
|  | Female | 953 | 49.76 | 8.425 |  |  |
| The presence of meaning, MLQ-P | Male | 172 | 24.72 | 4.478 | 1.835 | 0.067 |
|  | Female | 953 | 24.04 | 4.456 |  |  |
| The search for meaning, MLQ-S | Male | 172 | 26.35 | 5.477 | 1.467 | 0.143 |
|  | Female | 953 | 25.72 | 5.138 |  |  |
| Professional happiness | Male | 172 | 81.52 | 16.870 | 2.280 | **0.024** |
|  | Female | 953 | 78.39 | 14.906 |  |  |
| Physical and psychological health | Male | 172 | 18.12 | 5.889 | 3.628 | **＜0.001** |
|  | Female | 953 | 16.41 | 5.665 |  |  |
| Value or ability | Male | 172 | 21.51 | 4.780 | 3.434 | **0.001** |
|  | Female | 953 | 20.22 | 4.494 |  |  |
| Social support | Male | 172 | 20.05 | 3.750 | 1.336 | 0.182 |
|  | Female | 953 | 19.68 | 3.300 |  |  |
| Income | Male | 172 | 8.62 | 3.137 | -0.294 | 0.769 |
|  | Female | 953 | 8.69 | 2.823 |  |  |
| Working environment | Male | 172 | 13.22 | 3.366 | -0.654 | 0.513 |
|  | Female | 953 | 13.39 | 3.095 |  |  |

Appendix S2

| **Table S2 Multiple comparison by age groups**  **(**Dependent variable: turnover intention, LSD) | | | | | | |
| --- | --- | --- | --- | --- | --- | --- |
| (I)Group | (J) Group | Difference (I-J) | Standard error | *P* | 95% Confidence Intervals (95%CI) | |
|  |  |  |  |  | LCI | UCI |
| ＜30 | 30-40 | 0.297 | 0.115 | **0.010** | 0.07 | 0.52 |
|  | 41-51 | 0.783 | 0.551 | 0.155 | -0.30 | 1.86 |
| 30-40 | ＜30 | -0.297 | 0.115 | **0.010** | -0.52 | -0.07 |
|  | 41-51 | 0.486 | 0.549 | 0.376 | -0.59 | 1.56 |
| 41-51 | ＜30 | -0.783 | 0.551 | 0.155 | -1.86 | 0.30 |
|  | 30-40 | -0.486 | 0.549 | 0.376 | -1.56 | 0.59 |

| **Table S3 pairwise comparison by role**  **(**dependent variable: turnover intention, All pairwise) | | | | |
| --- | --- | --- | --- | --- |
| Role | Test statistic | Standard error | Standardized  test statistic | *P* |
| Administrative **vs.** Laboratory or radiology technician | 25.561 | 44.570 | 0.573 | 0.566 |
| Administrative **vs.** Researcher | 145.711 | 106.233 | 1.372 | 0.170 |
| Administrative **vs.** Nurse | 154.373 | 34.726 | 4.445 | **＜0.001** |
| Administrative **vs.** Physician | 218.683 | 43.201 | 5.062 | **＜0.001** |
| Laboratory or radiology technician **vs.** Researcher | -120.150 | 105.462 | -1.139 | 0.255 |
| Laboratory or radiology technician **vs.** Nurse | 128.813 | 32.291 | 3.989 | **＜0.001** |
| Laboratory or radiology technician **vs.** Physician | 193.122 | 41.270 | 4.680 | **＜0.001** |
| Researcher **vs.** Nurse | 8.663 | 101.694 | 0.085 | 0.932 |
| Researcher **vs.** Physician | 72.972 | 104.891 | 0.696 | 0.487 |
| Nurse **vs.** Physician | 64.309 | 30.374 | 2.117 | **0.034** |

| **Table S4 Multiple comparison by educational level**  **(**Dependent variable: turnover intention, LSD) | | | | | | |
| --- | --- | --- | --- | --- | --- | --- |
| (I) Level | (J) Level | Difference (I-J) | Standard error | *P* | 95% CI | |
|  |  |  |  |  | LCI | UCI |
| Associate degree or lower | Bachelor's degree | 0.003 | 0.375 | 0.993 | -0.73 | 0.74 |
|  | Master's degree | 0.457 | 0.390 | 0.242 | -0.31 | 1.22 |
|  | Doctor's degree or higher | -0.111 | 0.408 | 0.786 | -0.91 | 0.69 |
| Bachelor's degree | Associate degree or lower | -0.003 | 0.375 | 0.993 | -0.74 | 0.73 |
|  | Master's degree | 0.454 | 0.145 | **0.002** | 0.17 | 0.74 |
|  | Doctor's degree or higher | -0.114 | 0.187 | 0.543 | -0.48 | 0.25 |
| Master's degree | Associate degree or lower | -0.457 | 0.390 | 0.242 | -1.22 | 0.31 |
|  | Bachelor's degree | -0.454 | 0.145 | **0.002** | -0.74 | -0.17 |
|  | Doctor's degree or higher | -0.568 | 0.217 | **0.009** | -0.99 | -0.14 |
| Doctor's degree or higher | Associate degree or lower | 0.111 | 0.408 | 0.786 | -0.69 | 0.91 |
|  | Bachelor's degree | 0.114 | 0.187 | 0.543 | -0.25 | 0.48 |
|  | Master's degree | 0.568 | 0.217 | **0.009** | 0.14 | 0.99 |

| **Table S4 pairwise comparison by years in practice**  **(**dependent variable: turnover intention, All pairwise) | | | | |
| --- | --- | --- | --- | --- |
| Years | Test statistic | Standard error | Standardized test statistic | *P* |
| ＞20 **vs.** 16-20 | 64.919 | 82.788 | 0.784 | 0.433 |
| ＞20 **vs.** 11-15 | 156.339 | 77.273 | 2.023 | **0.043** |
| ＞20 **vs.** ≤5 | 222.174 | 75.055 | 2.960 | **0.003** |
| ＞20 **vs.** 6-10 | 255.777 | 74.769 | 3.421 | **0.001** |
| 16-20 **vs.** 11-15 | 91.420 | 45.573 | 2.006 | **0.045** |
| 16-20 **vs.** ≤5 | 157.255 | 41.701 | 3.771 | **＜0.001** |
| 16-20 **vs.** 6-10 | 190.858 | 41.184 | 4.634 | **＜0.001** |
| 11-15 **vs.** ≤5 | 65.835 | 29.262 | 2.250 | **0.024** |
| 11-15 **vs.** 6-10 | 99.438 | 28.521 | 3.486 | **＜0.001** |
| ≤5-6 **vs.** 10 | -33.603 | 21.806 | -1.541 | 0.123 |

| **Table S5 pairwise comparison by number of night shifts**  **(**dependent variable: turnover intention, All pairwise) | | | | |
| --- | --- | --- | --- | --- |
| Number | Test statistic | Standard error | Standardized test statistic | *P* |
| None **vs.** ＜5 | -99.644 | 28.675 | -3.475 | **0.001** |
| None **vs**. 5-9 | -148.296 | 27.987 | -5.299 | **＜0.001** |
| None **vs.** 10-15 | -163.918 | 32.006 | -5.121 | **＜0.001** |
| None **vs.** ＞15 | -264.459 | 49.240 | -5.371 | **＜0.001** |
| ＜5 **vs.** 5-9 | -48.652 | 24.685 | -1.971 | **0.049** |
| ＜5 **vs.** 10-15 | -64.274 | 29.163 | -2.204 | **0.028** |
| ＜5 **vs.** ＞15 | -164.815 | 47.441 | -3.474 | **0.001** |
| 5-9 **vs.** 10-15 | -15.621 | 28.487 | -.548 | 0.583 |
| 5-9 **vs.** ＞15 | -116.162 | 47.029 | -2.470 | **0.014** |
| 10-15 **vs.** ＞15 | -100.541 | 49.526 | -2.030 | **0.042** |
